# Leu432Val polymorphism in *CYP1B1* as a susceptible factor towards predisposition to primary open-angle glaucoma

**Published:** 2008-05-08

**Authors:** Ashima Bhattacharjee, Deblina Banerjee, Suddhasil Mookherjee, Moulinath Acharya, Antara Banerjee, Ananya Ray, Abhijit Sen, the Indian Genome Variation Consortium, Kunal Ray

**Affiliations:** 1 Molecular and Human Genetics Division, Indian Institute of Chemical Biology (Council of Scientific & Industrial Research), Kolkata, India; 2 Dristi Pradip, Jodhpur Park, Kolkata, India; 3 Nodal Laboratory, Institute of Genomics and Integrative Biology, New Delhi, India

## Abstract

**Purpose:**

Defects in *cytochrome P450 1B1* (*CYP1B1*) cause primary congenital glaucoma. However, defects in the gene have also been reported in primary open-angle glaucoma (POAG). Since POAG is primarily a complex disease, we examined the potential of coding single nucleotide polymorphisms (cSNPs) in the gene for association with the disease.

**Methods:**

Five coding SNPs – c.514 C>G (Arg48Gly), c.727 G>T (Ala119Ser), c.1666 C>G (Leu432Val), c.1719 C>T (Asp449Asp), and c.1730 A>G (Asn453Ser) – were genotyped in 264 unrelated POAG patients and 95 controls. In addition, 542 normal individuals selected from various ethnic groups representing the Indian population were also genotyped for these cSNPs. The patterns of linkage disequilibrium between the SNPs and haplotype variations for comparison between POAG patients and controls as well as different ethnic groups of the Indian population were determined using Haploview. Allelic variants of Leu432Val were cloned by site-directed mutagenesis of normal *CYP1B1* cDNA, which were used for transfection of retinal pigment epithelium (RPE) cells. The generation of reactive oxygen species (ROS) was quantified by measuring fluorescence emission by degradation of CM-H2DCFDA using a fluoremeter.

**Results:**

The c.1666G allele of the Leu432Val in *CYP1B1* showed a statistically significant higher representation among POAG patients compared to controls (p=0.0001; Odds ratio=6.027; 95% CI: 3.863–9.401) suggesting it to be a potential risk allele toward disease predisposition. Analysis of genotype frequencies of the polymorphism between the two groups demonstrated GG as a potential risk genotype (p=0.0001; Odds ratio=15.505; 95% CI: 5.529–43.474) for the disease. CYP1B1 Val432 was estimated to generate higher ROS in RPE cells compared to its allelic variant (Leu432; p=0.0245 for 15 min and p=0.0197 for 30 min). Comparison of haplotype diversities revealed CGGTA as the risk haplotype for the disease (p=0.0001, by Fisher’s exact test).

**Conclusions:**

We report *CYP1B1* c.1666G (Val432) as a susceptible allele for POAG and CGGTA as the risk haplotype for the disease. Higher ROS generation by Val432 in CYP1B1 might lead to apoptotic change that leads to glaucoma. Remarkable variation of the cSNPs observed among ethnic groups of India could provide insight for future epidemiological studies on POAG in these population groups.

## Introduction

Primary open-angle glaucoma (POAG) is the most common form of glaucoma. Among 14 implicated chromosomal loci (GLC1A – GLC1N) [1-12], three underlying candidate genes have been identified – *myocilin (MYOC)*, *optineurin (OPTN)*, and *WD40-repeat 36* (*WDR36*) [2,13,14]. Recent studies suggest that POAG is caused mainly by genetic predisposition and interaction with other risk factors [15]. The study, which is based on published literature, estimated that 72% of all POAG cases represent the inherited or familial form of the disease that does not show a clear pattern of Mendelian inheritance.

Among genes implicated to have a potential role in POAG causation, *cytochrome P450 1B1 *(*CYP1B1*) poses as an interesting candidate for investigation. CYP1B1, a member of the cytochrome p450 family of monooxygenases, is also widely known for its role in steroid metabolism and xenometabolic detoxification [16]. Defects in *CYP1B1* cause an autosomal recessive form of primary congenital glaucoma (PCG) [17,18]. In addition, recent studies indicate the gene’s role in anterior segment dysgenesis like Peters’ anomaly [18]. The gene has been found to be involved in expediting disease onset in a familial case of open-angle glaucoma when present alongside a heterozygous mutation in *MYOC*. Therefore, the gene acts as a modifier locus [19]. Moreover, *CYP1B1* has recently been shown to have primary involvement in a familial case of juvenile onset POAG [20,21], and missense mutations have been detected in sporadic POAG cases that are absent in controls [21,22].

In addition to the reported involvement of missense mutations in *CYP1B1* with different forms of glaucoma, coding single nucleotide polymorphisms (cSNPs) within the gene have been found to be associated with a predisposition for complex diseases like different types of cancer (viz., breast, lung, prostate, and endometrial cancer) [23-25]. A study performed on *CYP1B1* SNPs in French POAG patients has reported an association of a common coding polymorphism (Asn453Ser) with glaucomatous clinical features such as optic disc cupping and visual field alteration [26]. The present study investigates the role of coding SNPs detected in *CYP1B1* in the POAG patient pool from Eastern India for their association as risk factors toward POAG predisposition. This study also attempts to investigate the effect of the Leu432Val polymorphism in *CYP1B1* on a generation of superoxide species as a potential cause of neurodegeneration in POAG.

## Methods

### Selection of study subjects

A group of 264 Indian POAG patients residing in West Bengal (Eastern India) and speaking Bengali were recruited from the Dristipradip Eye Clinic, Kolkata, India regardless of their status of family history of POAG. Diagnoses involved clinical, ocular, and systemic examinations. Intraocular pressure (IOP) was initially measured by air puff non-contact tonometer. A Goldman 3-mirror gonioscope (Ocular Instrument, Bellevue, WA)was used to assess the angles of the anterior chamber and optic disc. The optic disc was also evaluated with a +78D lens in some patients. Automated threshold field analysis was done using the Humphrey Field Analyzer II (Carl Zeiss, Dublin, CA) or the Medmont M600 Automated Perimeter (Medmont, Camberwell, Victoria, Australia). The retinal nerve fiber layer (RNFL) was investigated by scanning laser polarimetry with variable corneal compensation technique. For glaucoma cases identified by ocular examinations mentioned above, IOP was reassessed by Goldmann applanation tonometry (Haag-Streit USA Inc., Mason, OH) followed by pachymetry.

An increased intraocular pressure above 21 mmHg, significant cupping of the optic disc with or without peripapillary changes, and the presence of an open angle of the anterior chamber raised the suspicion of POAG, which was confirmed by typical reproducible visual field changes in an automated perimetry test. Individuals who had an IOP of less than 21 mmHg but had cupping of the optic disc and visual field changes characteristic of POAG were also included in the study. Thus, the patient pool consisted of 37 juvenile onset open-angle glaucoma cases (ages 10–35 years) and 227 adult onset open-angle glaucoma cases. The age at diagnosis ranged from 10 to 84 years with a mean±standard deviation of 55.69±16.78 years. However, individuals with any history of inflammation or ocular trauma (past and present) and ocular hypertension were excluded from this study. Ninety-five ethnically matched controls were also recruited in this study. They were determined to be negative for POAG based on a routine eye examination for glaucoma including direct ophthalmoscopy, a thorough examination of the optic disc and intraocular tension, gonioscopy, automated visual field analysis, and retinal nerve fiber layer (RNFL) analysis with the help of scanning laser polarimetry (SLP) with variable corneal compensation.

**Figure 1:**
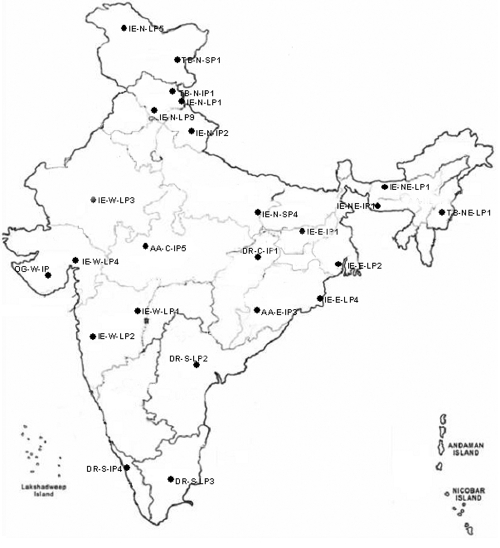
Map of India showing the respective locations of the 24 ethnic groups representing the population of the country. These ethnic groups are classified on the basis of four major linguistic groups (Indo-European, Austro-Asiatic, Tibeto-Burman, and Dravidian) that define genetic variation in the population based on reported studies conducted on mitochondrial and autosomal genes.

For study of the Leu432Val polymorphism in *CYP1B1* in the general Indian population as shown in Figure 1, the study subjects consisted of 542 individuals from 24 ethnic subgroups (each consisting of 20–23 individuals; Table 1) classified on the basis of four major linguistic groups (Indo-European, Austro-Asiatic, Tibeto-Burman, and Dravidian) that define genetic variation in the population based on studies conducted on mitochondrial [27] and autosomal [28,29] genes.

**Table 1:** Ethnic groups representing the general Indian population.

**Ethnic groups**	**Linguistic affiliation**	**Number of individuals**	**Geographical region**
IE-E-IP1	Indo-European	23	East
IE-E-LP2	Indo-European	23	East
IE-NE-IP1	Indo-European	23	North-east
IE-N-LP9	Indo-European	22	North
IE-N-LP1	Indo-European	22	North
IE-W-LP1	Indo-European	23	West
IE-W-LP2	Indo-European	23	West
IE-W-LP3	Indo-European	22	West
IE-N-LP5	Indo-European	22	North
IE-NE-LP1	Indo-European	23	North-east
IE-E-LP4	Indo-European	23	East
IE-W-LP4	Indo-European	23	West
IE-N-SP4	Indo-European	23	North
IE-N-IP2	Indo-European	23	North
DR-C-IP1	Dravidian	22	Central
DR-S-LP3	Dravidian	23	South
DR-S-IP4	Dravidian	23	South
DR-S-LP2	Dravidian	23	South
TB-N-IP1	Tibeto-Burman	23	North
TB-NE-LP1	Tibeto-Burman	22	North-east
TB-N-SP1	Tibeto-Burman	23	North
AA-C-IP5	Austro-Asiatic	20	Central
AA-E-IP3	Austro-Asiatic	22	East
OG-W-IP	Austro-Asiatic	23	West

The ethnic groups are described in the first column by their linguistic affiliation, geographical location, and population size in abbreviated form as follows: Linguistic groups- IE, Indo European; DR, Dravidian; TT, Tibeto Burmese; AA, Austro Asiatic; and OG, Out Groups; Geographical locations- C, Central; E, Eastern; N, North; NE, Northeastern; and S, South; Population types- IP, Isolated Population; LP, Large population; and SP, Small population.

### Collection of blood samples and genomic DNA preparation

Ten milliliters of peripheral blood was collected with EDTA from the POAG patients and normal individuals with their written consent. For the study of intragenic SNPs in *CYP1B1* in different linguistic groups of the Indian subcontinent, populations were identified and samples were collected with the help of trained anthropologists, social workers, and community health workers. Endogamy of the populations was established by gathering extensive information about the marriage patterns from pedigrees and interview of family members of the donor as well as published literature. It was ensured that the individuals were unrelated at least to the first cousin level, and attempts were made to collect blood samples from both males and females in equal numbers.

Genomic DNA was prepared from fresh whole blood using the conventional phenol chloroform method followed by ethanol precipitation. Then, the DNA was dissolved in TE (10 mM Tris-HCl, 0.1 mM EDTA, pH 8.0) [30]. The study protocol adhered to the tenets of the Declaration of Helsinki and was approved by the Institutional Review Board.

### Polymerase chain reaction and DNA sequencing

A polymerase chain reaction (PCR) was performed in a total volume of 25 μl containing 50–100 ng genomic DNA to amplify *CYP1B1* exons and adjoining splice junctions as described in reference [20]. The specific PCR products were subjected to bidirectional sequencing using an ABI 3130XL DNA sequencer (Applied Biosystems, Foster City, CA) with dye termination chemistry to identify any alteration of sequence. The selected cSNPs in different linguistic groups of the Indian population were identified using homogeneous MassEXTEND (hME) assay, an effective genotyping method run on the MassARRAY system, done using SEQUENOME (San Diego, CA) as described in reference [31].

### Bioinformatic and statistical analysis

Haplotypes were determined for comparison between patients and controls using Haploview 3.2 software. Allele frequencies of the cSNPs were compared between patients and controls using the χ^2^ test. A generation of reactive oxygen species (ROS) was compared between the *CYP1B1* allelic variants using the unpaired Student’s *t*-test.

### Site-directed mutagenesis

A *CYP1B1* cDNA construct in the pcDNA3 mammalian expression vector (kindly supplied by Dr. Thomas H. Friedberg, University of Dundee, Dundee, Scotland) was used to generate a clone having a C to G change at the 1,666^th^ nucleotide. The oligonucleotides used for this purpose were 5′– GTC TGT GAA TCA TGA CCC AGT GAA GTG GCC TAA CCC GGA G –3′ and 5′– CTC CGG GTT AGG CCA CTT CAC TGG GTC ATG ATT CAC AGA CC –3′. Site-directed mutagenesis and the subsequent transformation of the mutant clone were performed using the QuikChange XL Site-Directed Mutagenesis Kit (Stratagene, La Jolla, CA) according to the manufacturer’s protocol. Plasmid isolation was done for both the c.1666C and c.1666G variants cloned in the pcDNA3 vector using a Qiagen plasmid mini kit following the protocol provided by the manufacturer (Qiagen, Hilden, Germany), and the generation of the mutant clone was confirmed by sequencing the inserts within the mutagenized recombinant clone. The insert containing *CYP1B1* cDNA in both the original and mutant clones was entirely sequenced. It was confirmed that both the sequences were identical except the intended mutagenized base (c.1666C>G).

### Mammalian cell culture and transfection

The human retinal pigment epithelium cell line, RPE8319 (a kind gift from Dr. Frans Cremers, University Medical Center, Nijmegen, the Netherlands), was maintained, and transfections were performed following the protocol described previously in reference [32]. Cells were harvested after 42 h of transfection for the detection of reactive oxygen species (ROS).

### Detection and comparison of the generation of reactive oxygen species

Cells were transfected with recombinant *CYP1B1* clones with either the Leu432 or Val432 variant. Forty-two hours after transfection, cells were trypsinized, washed with PBS, kept in DMEM containing 10% FBS for 1 h, and treated with 17β-estradiol. Approximately equal numbers of transfected, untransfected, and H_2_O_2_-treated cells were incubated with 20 µM 5-(and-6)-chloromethyl-2'7'-dichlorodihydrofluorescein diacetate acetyl ester (CM-H2DCFDA) at 37 °C for 30 min. Fluorescence was measured through a spectrofluorometer by using 507 nm as the excitation wavelength and 530 nm as the emission wavelength. Basal fluorescence was subtracted from all measurements.

## Results

Analysis of the coding regions in 264 POAG patients led to the identification of five coding polymorphisms, Arg48Gly, Ala119Ser, Leu432Val, Asp449Asp, and Asn453Ser in addition to six mutations identified earlier in nine POAG patients [20].

### Linkage disequilibrium pattern between the coding single nucleotide polymorphisms

**Figure 2:**
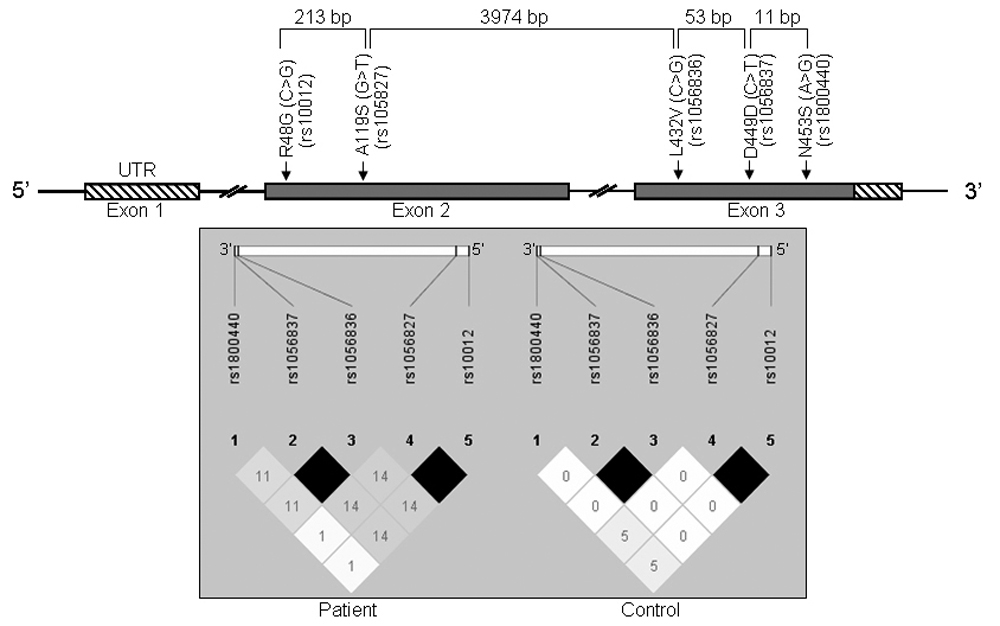
Linkage disequilibrium (LD) pattern (r^2^) of the five coding single nucleotide polymorphisms in *CYP1B1* in primary open-angle glaucoma patients and controls. The extent of LD lowers as the shading gets lighter (calculated with Haploview 3.2 using standard color schemes). It is to be noted that the direction (5′ to 3′) of the cSNPs in the top panel (cartoon of the gene) and the bottom panel (LD pattern) is reversed.

Out of five cSNPs analyzed in *CYP1B1*, Arg48Gly and Ala119Ser, as well as Leu432Val and Asp449Asp were found to be in perfect linkage disequilibrium (LD; r^2^ value 1), which was calculated using the Haploview program (Figure 2). Hence, three out of the five cSNPs (Arg48Gly, Leu432Val, and Asn453Ser) were selected for further study among patients and controls.

### Leu432Val as a risk factor for primary open-angle glaucoma predisposition

Comparison of the allele frequencies of the three aforementioned cSNPs among patients and controls revealed biased distribution for the Leu432Val polymorphism with c.1666G (Val432) showing significantly higher representation (p=0.0001) among POAG patients (Table 2) than in control individuals. Our observation suggests c.1666G (Val432) as the “risk allele” toward POAG predisposition (Odds ratio=6.027; 95% CI: 3.863 – 9.401) and GG as a “risk genotype” (p=0.0001; Odds ratio=15.505; 95% CI: 5.529 – 43.474). A similar observation was made when the sample pool was analyzed separately for normotensive and hypertensive groups.

**Table 2:** Allele frequencies of the *CYP1B1* cSNPs in primary open-angle glaucoma patients and controls.

**Sample**	**R48G (C>G)**			**L432V (C>G)**			**N453S (A>G)**		
	**C**	**G**	**Total (chromosomes)**	**C**	**G**	**Total (chromosomes)**	**A**	**G**	**Total (chromosomes)**
**Case**	0.68 (358)	0.32 (170)	528	0.51 (270)	0.49 (258)	528	0.87 (461)	0.13 (67)	528
**Control**	0.64 (122)	0.36 (68)	190	0.86 (164)	0.14 (26)	190	0.89 (170)	0.11 (20)	190

Comparison of the allele frequencies of the three cSNPs among cases (patients) and controls revealed biased distribution for the Leu432Val polymorphism. c.1666G (Val432) showed significantly higher representation (p=0.0001) in POAG patients than controls.

### *CYP1B1* coding single nucleotide polymorphisms in different ethnic groups of the Indian population

Based on the LD patterns of *CYP1B1* cSNPs discussed earlier, allele and genotype frequencies of the three cSNPs in *CYP1B1*,Arg48Gly, Leu432Val, and Asn453Ser were studied in 542 individuals representing 24 ethnic groups of the Indian population.

**Figure 3:**
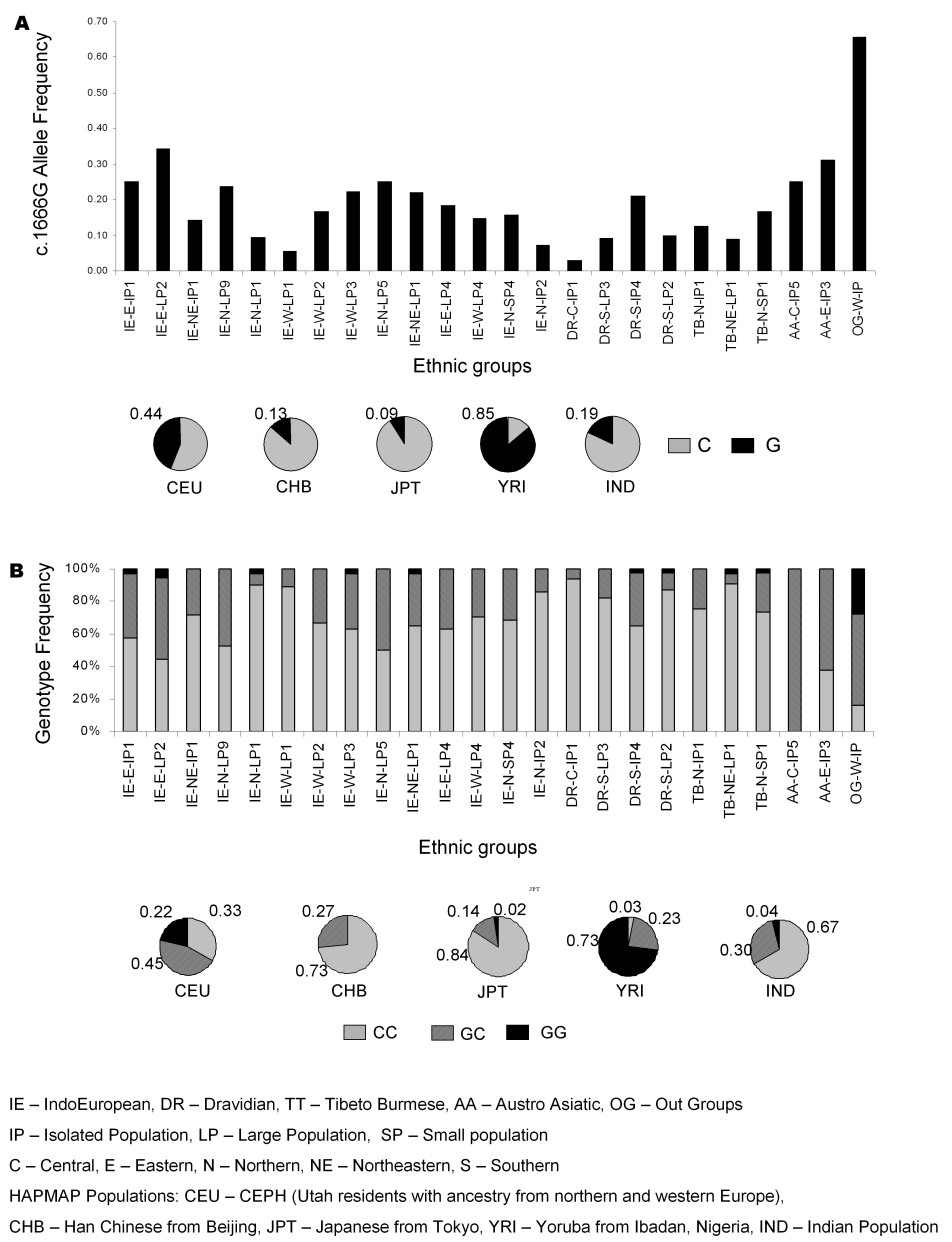
Allele and genotype frequency distribution of the Leu432Val polymorphism in *CYP1B1* in different ethnic groups of Indian and HapMap populations. **A**: Allele frequency of various ethnic groups is shown by bar diagram while the overall allele frequency among Indians as well as populations included in HapMap project are shown by pie chart. **B**: Similarly, all three genotype frequencies in various ethnic groups of India and the populations included in HapMap are shown by bar diagram and pie chart, respectively. The following are the meanings of each abbreviation of the linguistic groups: IE, Indo European; DR, Dravidian; TT, Tibeto Burmese; AA, Austro Asiatic; OG, Out Group population types; IP, Isolated Population; LP, Large population; and SP, Small population. The following are abbreviations for geographical locations: C, Central; E, Eastern; N, North; NE, Northeastern; and S, South. The following are the abbreviations for HapMap populations: CEU, CEPH (Utah residents with ancestry from northern and western Europe); CHB, Han Chinese from Beijing; JPT, Japanese from Tokyo; YRI, Yoruba from Ibadan, Nigeria; and IND, Indian population.

Leu432Val showed variable minor allele frequencies across different ethnic groups (Figure 3). However, it is noteworthy that c.1666G (Val432) was found to be the major allele in the OG-W-LP5 ethnic group like Yoruba (YRI) with the highest frequency of the risk genotype (GG) among all the population groups. Interestingly, OG-W-LP5 is regarded as an out-group of African descent. Detailed epidemiological information on the prevalence of glaucoma in different parts of the country especially in the OG-W-LP5 population and the correlation of disease prevalence with frequency of the c.1666G allele in the *CYP1B1* gene can shed light on the possible involvement of the variant in disease causation.

### Functional analysis of the Leu432Val variant of CYP1B1

Leu432Val is reported to affect the catalytic property of CYP1B1. Li et al. [33] reported that the Val432 form of CYP1B1 displayed a fourfold lower K_m_ compared to the Leu432 form. The metabolite, 4-hydroxyestradiol, is a potent carcinogen resulting from 17β-estradiol. This metabolite is known to undergo redox cycling with the formation of reactive quinones, which can create oxidative stress [34]. In the presence of estradiol, CYP1B1 having Val at position 432 is expected to generate a higher amount of reactive superoxides in cells compared to the enzyme having Leu at position 432.

**Figure 4:**
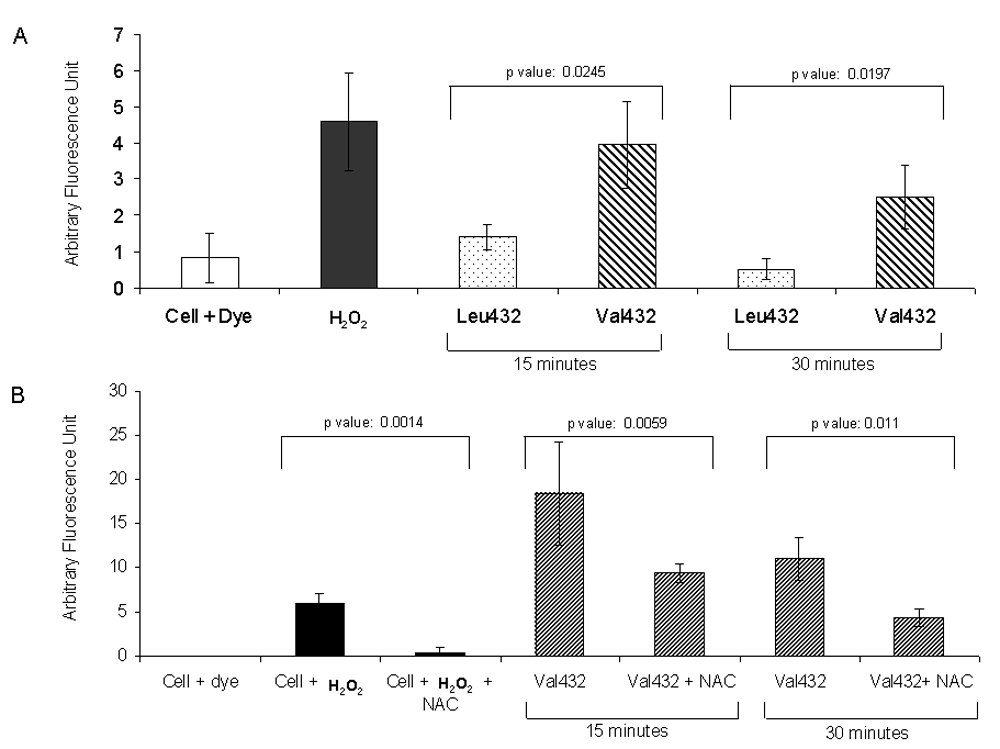
Comparison of reactive oxygen species generation in retinal pigment epithelium cells transfected with *CYP1B1* variants of Leu432Val polymorphism. **A**: Cells were transfected with recombinant *CYP1B1* clones with either the Leu432 or Val432 variant, trypsinized, washed with PBS, and treated with 400 nM 17β-estradiol. Approximately equal numbers of transfected, untransfected, and H_2_O_2_-treated cells (used as positive control) were incubated with CM-H2DCFDA and fluorescence was measured as described in Methods section. Basal fluorescence was subtracted from all measurements. **B**: To substantiate the generation of fluorescence due to ROS, cells subjected to similar treatment as that of panel **A** were further treated with or without 2 mM NAC (n-acetyl cysteine) before treatment with 17β-estradiol. As seen in the figure a significant decrease in fluorescence in NAC-treated cells were observed compared to the untreated ones.

To investigate this, retinal pigment epithelium (RPE) cells transfected with recombinant clones of *CYP1B1* having either the Val432 or Leu432 variant were treated with 17β-estradiol in a time-dependent (15 and 30 min) and dose-dependent (400 and 600 nM) manner to determine the optimum conditions (400 nM). Cells treated with 25 µM H_2_O_2_ were kept as a positive control (Figure 4) for the assay. The fluorescence intensity estimates for cells transfected with Leu432 or Val432 showed higher ROS generation (p=0.0245 for 15 min and p=0.0197 for 30 min) in cells transfected with the Val432 variant at both time points (Figure 4).

To further substantiate the generation of fluorescence due to ROS, cells were incubated with 2 mM n-acetyl cysteine (NAC), which is known to scavenge superoxides, before treatment with 17β-estradiol. The results showed a significant decrease in fluorescence in NAC-treated cells compared to the untreated ones (Figure 4), validating that the fluorescence detected is due to the generation of superoxides. By DNA sequencing, we ensured that the *CYP1B1* cDNA insert in the mutant clone (c.1666 G variant) did not harbor any other base change that could have altered the biologic activity relative to the wild type clone (c.1666 C variant).

### *CYP1B1* haplotype variation among primary open-angle glaucoma patients and controls

**Figure 5:**
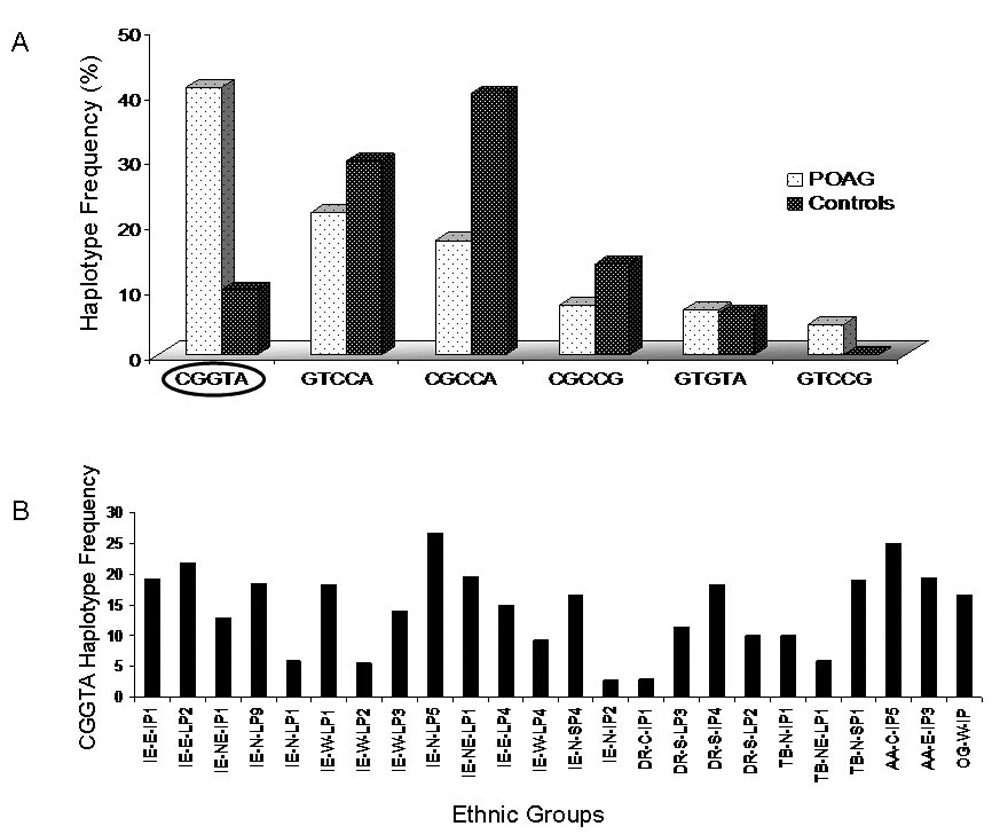
*CYP1B1* haplotype distribution. **A**: *CYP1B1* haplotype variation among primary open-angle glaucoma patients and controls The Study of haplotype diversity among patients and controls revealed C-G-G-T-A as the predominant haplotype among POAG patients (41%) and a lower representation among controls (10%). Hence, the haplotype represents a potential risk haplotype among POAG patients. **B**: The distribution of the risk haplotype (CGGTA) in 24 ethnic groups of the Indian population. The study on the distribution of the risk haplotype (C-G-G-T-A) in the various ethnic groups of the Indian population revealed the highest frequency (26.6%) among the ethnic group, IE-N-LP5, followed by AA-C-IP5 (25%). In the OG-W-LP5 group where c.1666G (Val432) is the major allele, the risk haplotype frequency was estimated to be 16.7%.

Haplotypes were constructed using Haploview with the studied cSNPs in the following order: c.514 C>G, c.727 G>T, c.1666 C>G, c.1719 C>T, and c.1730 A>G. Study of haplotype diversity (Figure 5) among patients and controls revealed C-G-G-T-A as the predominant haplotype among POAG patients (41%) and a lower representation among controls (10%; p=0.0001, by Fisher’s exact test; Table 3). Hence, the haplotype represents a potential risk haplotype among POAG patients. Interestingly, this haplotype has been reported to harbor a vast majority of the *CYP1B1* mutations causal to primary congenital glaucoma (PCG) across different population groups worldwide [35]. Therefore, the risk haplotype for POAG as revealed by our study also represents the risk haplotype for PCG. However, among the nine POAG patients harboring *CYP1B1* mutations [20], we could determine haplotype in only two patients with access to family members’ samples, but the risk haplotype (C-G-G-T-A) was not identified in either of them. Incidentally, we observed that the haplotype of the wild type *CYP1B1* cDNA clone (c.1666 C variant), used to examine functional variation of Leu432Val polymorphism, is the same as the most common haplotype (C-G-C-C-A) present in 40% of the controls examined (Table 3). The risk haplotype (C-G-G-T-A), which is overrepresented in the POAG patients, is functionally equivalent to the haplotype (C-G-G-C-A), which is present in the mutant clone, since the cSNP (c.1719 C>T) in LD with Leu432Val (c.1666 C>G) polymorphism codes for a synonymous change (Asp449Asp).

**Table 3:** Haplotype diversity of *CYP1B1* coding single nucleotide polymorphisms among primary open-angle glaucoma patients and controls

**SI number**	**Haplotype**	**POAG patients (%)**	**Controls (%)**
1	C-G-G-T-A	41	10
2	G-T-C-C-A	21.8	29.7
3	C-G-C-C-A	17.5	40
4	C-G-C-C-G	7.5	13.9
5	G-T-G-T-A	6.8	6.4
6	G-T-C-C-G	4.6	0

Haplotypes were constructed using cSNPs in the following order: c.514 C>G, c.727 G>T, c.1666 C>G, c.1719 C>T, and c.1730 A>G. Among major haplotypes, C-G-G-T-A is overrepresented in POAG patients (41%) compared to controls (10%; p=0.0001, by Fisher’s exact test). Hence, C-G-G-T-A represents a potential risk haplotype among POAG patients.

### Distribution of the risk haplotype throughout the Indian population

As shown in Figure 5B, the study on the distribution of the risk haplotype (C-G-G-T-A) in the various ethnic groups of the Indian population revealed the highest frequency (26.6%) among the ethnic group, IE-N-LP5, followed by AA-C-IP5 (25%). In the OG-W-LP5 group where c.1666G (Val432) is the major allele, the risk haplotype frequency was estimated to be 16.7%.

## Discussion

POAG is primarily a complex disease showing sporadic occurrence in the population and is thought to involve interplay between multiple genes and the environment. While screening for mutations, we also detected SNPs in *CYP1B1* of POAG patients. SNPs play a major role in susceptibility to complex diseases through their subtle effects on the protein. A nonsynonymous SNP (Asn453Ser) in *CYP1B1* has been reported to be associated with clinical features like optic disc cupping and visual field alteration in French POAG patients [26].

The significant difference reported here in the distribution of the allele frequency of the c.1666G (Val432) allele of Leu432Val and corresponding GG genotype among patients and controls points toward it being a risk allele for the disease. Evidence from the literature indicates that the variant has been implicated in head and neck squamous cell carcinoma (HNSCC) where smokers with HNSCC having the GG genotype are 20 times more likely to show *p53* mutations compared to individuals with the CC genotype [36]. Their data indicates a strong and consistent association between the Leu432Val polymorphism in *CYP1B1* and the smoke-induced *p53* mutations. The bias observed in the distribution of the allele and genotype frequency of the Leu432Val prompted us to look for variation in the haplotype diversities between the two groups. Our initial observation that CGGTA serves as the potential risk haplotype for POAG has been recently supported by another study [37] and represents the same haplotype reported as a risk factor for PCG [35].

The reported effect of the Val432 variant in CYP1B1 on higher enzyme activity [33] and the carcinogenic effects of the downstream metabolite through quinone formation [34] led us to explore the role of the variant on the generation of superoxides. Data on the ROS generation in RPE cells following transfection with *CYP1B1* variants and 17β-estradiol treatment shows a higher generation of ROS in cells having the Val432 variant in the CGGCA haplotype background than cells having the Leu432 variant in the CGCCA background. Therefore, it is likely that the mutant CYP1B1 would similarly affect trabecular meshwork and retinal ganglion cells involved in POAG pathogenesis. However, the observation needs to be confirmed in these cell lines by replicating the same experiment or similar studies. It is noteworthy that reactive superoxides generated in a non-cycling cell population can lead to apoptosis, cell death, and degeneration [38]. Hence, such generation of superoxides over a long period of time can drive ocular cells to apoptosis, and reports suggest oxidative stress to be one of the major causes of apoptotic loss of retinal ganglion cells in glaucoma [38]. Therefore, inter-individual differences in estrogen metabolism resulting from the Leu432Val variant in CYP1B1 may lead to differences in individual susceptibility to complex diseases like POAG. In a reported molecular model of CYP1B1 [39], the 432^nd^ residue is located in a large meander region structurally close to Lys454. The latter residue is structurally equivalent to Arg422 in CYP2B4, identified to be involved in ionic interactions with cytochrome P450 reductase [33,40].

Comparison of the allele frequencies of the Leu432Val polymorphism in the four world populations enlisted in the HAPMAP database (Figure 3) showed Leu432 as the major allele in Caucasians (CEU) and Asians (CHB and JPT) while Val432 is the major allele among the Yuroba (YRI) from Nigeria. Interestingly, epidemiological studies suggest a higher incidence of glaucoma among Africans and Afro-Americans compared to Caucasians [41,42]. Thus, it is worth examining the association of the *CYP1B1* variant with POAG among African and/or Afro-Americans with incidence of glaucoma similar to that performed in prostate cancer [43]. Allele frequency distribution of the Leu432Val polymorphism across different subpopulations showed variable frequency of the minor allele with the risk allele being the major allele in OG-W-LP5, which is similar to that observed in Yoruba.

The present study reports for the first time the association of the Leu432Val polymorphism in *CYP1B1* as a risk factor for POAG, which is further supported by the in vitro functional analysis. Further, information gained on the genetic variation of this polymorphism among different ethnic groups of India could be helpful for future epidemiological studies on the prevalence of POAG among Indian subpopulations. Such effort toward disease gene exploration has been recently reported by Indian Genome Variation Consortium [44]. The risk haplotype (CGGTA) represents one of the two ancestral *CYP1B1* haplotypes [35]. It would be interesting to study the distribution of this haplotype with the simultaneous assessment of its involvement in POAG predisposition in different world populations.
